# Design and rationale of the South-East Netherlands Heart Registry (ZON-HR)

**DOI:** 10.1007/s12471-025-01934-7

**Published:** 2025-02-06

**Authors:** Eva C. I. Woelders, Denise A. M. Peeters, Sanne Janssen, Jasper J. P. Luijkx, Patty J. C. Winkler, Peter Damman, Wouter S. Remkes, Arnoud W. J. van ’t Hof, Robert Jan M. van Geuns, ZON-H.R. investigators, E. C. I. Woelders, D. A. M. Peeters, P. Damman, R. J. M. van Geuns, N. van Royen, M. H. van Wely, C. Camaro, T. ten Cate, A. C. Dimitriu-Leen, L. X. van Nunen, S. Janssen, J. J. P. Luijkx, A. W. J. van ’t Hof, P. J. C. Winkler, M. Ilhan, A. W. Ruiters, A. Lux, M. Stein, S. Rasoul, R. A. L. J. Theunissen, J. Vainer, L. F. Veenstra, P. A. Vriesendorp, L. P. C. Hoebers, K. Kulekci, A. J. J. Ijsselmuiden, W. S. Remkes, S. Aydin, B. M. Rahel, F. L. J. Eerens, V. V. Hemradj

**Affiliations:** 1 Department of Cardiology, Radboud University Medical Centre, Nijmegen, The Netherlands; 2 Department of Cardiology, Zuyderland Medical Centre, Heerlen, The Netherlands; 3 Department of Cardiology, VieCuri Medical Centre, Venlo, The Netherlands; 4 Department of Cardiology, Maastricht University Medical Centre+, Maastricht, The Netherlands; 5 Cardiovascular Research Institute Maastricht (CARIM), Maastricht, The Netherlands

**Keywords:** Percutaneous coronary intervention, Chronic coronary syndrome, Acute coronary syndrome, Secondary prevention, Personalised antiplatelet strategy

## Abstract

**Introduction:**

In patients undergoing percutaneous coronary intervention (PCI), personalised medicine is key to the secondary prevention of ischaemic and bleeding events. To provide an extensive overview of the quality of secondary prevention and of personalised medicine, a consortium in the southeastern region of the Netherlands has created a PCI registry: the South-East Netherlands Heart Registry (*Zuid-Oost Nederland Hart Registratie*, ZON-HR).

**Aim:**

To visualise and improve personalised secondary prevention post-PCI, focussing on key elements such as antiplatelet treatment, cholesterol management and comorbidities such as diabetes mellitus.

**Design and population:**

A prospective multicentre registry of all consecutive patients undergoing PCI at 4 participating PCI centres and 3 referral centres.

**Treatment:**

Interventional procedures and concomitant pharmaceutical treatment are performed in accordance with the guidelines. The ZON-HR promotes risk stratification after PCI using a simplified protocol for a personalised antiplatelet strategy.

**Data collection and quality:**

Demographics, laboratory values, baseline procedural characteristics and pharmaceutical treatment data are collected. Outcomes include thromboembolic and bleeding complications and medication changes. Data are pseudonymised, and a clinical event committee will review 20% of the adverse events (randomly selected).

**Strengths and weaknesses:**

This registry represents the entire PCI population and visualises gaps in secondary prevention. Weaknesses are the collection of outcomes and medication changes using mostly patient-reported outcomes.

**Conclusion:**

The ZON-HR is a comprehensive PCI registry that provides baseline and follow-up data of a large PCI cohort in the southeastern region of the Netherlands. The ZON-HR aims to improve secondary prevention after PCI and augment personalised treatment that focusses on key elements of secondary prevention.

**Supplementary Information:**

The online version of this article (10.1007/s12471-025-01934-7) contains supplementary material, which is available to authorized users.

## Introduction

Patients who have undergone a percutaneous coronary intervention (PCI) for chronic coronary syndrome (CCS) or acute coronary syndrome (ACS) have a residual risk of ischaemic events due to the progression of atherosclerosis in and outside the stented segments [1, 2]. Secondary prevention after PCI is therefore of major importance to reduce complications and improve their prognosis.

Antithrombotic treatment plays a major role in the reduction of thromboembolic events and is a key part of secondary prevention. However, this treatment increases the risk of bleeding complications. Multiple studies have demonstrated that a patient-tailored approach for antiplatelet therapy based on thromboembolic and bleeding risk factors minimises these complications [2–8]. Therefore, a personalised antiplatelet strategy is recommended by clinical guidelines [9–12]. In combination with antithrombotic treatment, lipid management is the cornerstone of secondary prevention. As low-density lipoprotein cholesterol (LDL-C) increases the risk for atherosclerotic cardiovascular disease, the current European Society of Cardiology (ESC) Guidelines recommend LDL-C < 1.4 mmol/l in patients undergoing PCI [13–15]. Personalised medicine in which post-PCI treatment and lifestyle advice are adjusted based on the patient’s risk factors such as high bleeding or thromboembolic risk, high LDL‑C levels, hypertension or the presence of diabetes mellitus (DM) is of major importance to lower residual risks of complications and repeat revascularisation. Although the guidelines recommend personalised medicine, this is challenging due to the large number of risk factors that should be considered. Therefore, adherence to these recommendations is challenging, which can lead to undertreatment.

To maintain and improve the quality of care throughout the Netherlands, all interventional centres are mandated to submit data on patient and procedural characteristics and follow-up after PCI to the Netherlands Heart Registry (NHR). The NHR processes the data and provides these centres with feedback [16]. However, the registration of baseline, procedural and follow-up parameters by the NHR is concise and mainly focuses on mortality and (ischaemia-driven) revascularisation. To enhance the potency of this PCI registry, a consortium in the southeastern region of the Netherlands has created an extended version: the South-East Netherlands Heart Registry (*Zuid-Oost Nederland Hart Registratie*, ZON‑HR). This comprehensive registry provides a more extensive overview of the quality of secondary prevention and personalised medicine. Furthermore, the ZON-HR Consortium aims to improve secondary prevention post-PCI by stimulating a personalised approach. In addition, it will provide a platform for research on secondary prevention.

## Methods

### Registry design

The ZON-HR is a prospective multicentre registry of all consecutive patients with coronary artery disease at 4 participating PCI centres and 3 referral centres in the southeastern region of the Netherlands who undergo clinically indicated PCI at one of the following participating centres: Radboud university medical centre, Maastricht University Medical Centre, Zuyderland Medical Centre and VieCuri Medical Centre. Annually, approximately 3500 patients undergo PCI at one of these participating centres, of whom two-thirds have ACS. Of all the patients in the ZON-HR, 20% come from referral centres.

Data collection started in November 2020 (at the first centre) and is ongoing. Data are collected in Castor Electronic Data Capture (EDC) [17] in a pseudonymised manner. This registry was evaluated by the medical ethics committee Zuyderland & Zuyd. As patients are not subjected to any procedures other than those recommended in the guidelines and protocols and because the main purpose of this registry is quality control, the registry is not subject to the Dutch Medical Research Involving Human Subjects Act (*Wet medisch-wetenschappelijk onderzoek met mensen*). Therefore, no informed consent is required. However, patients are offered an opt-out option via follow-up questionnaires. The study protocol conforms to the ethical guidelines of the 1975 Declaration of Helsinki and is registered at ClinicalTrials.gov (NCT06512493).

### Study population

**Fig. 1:**
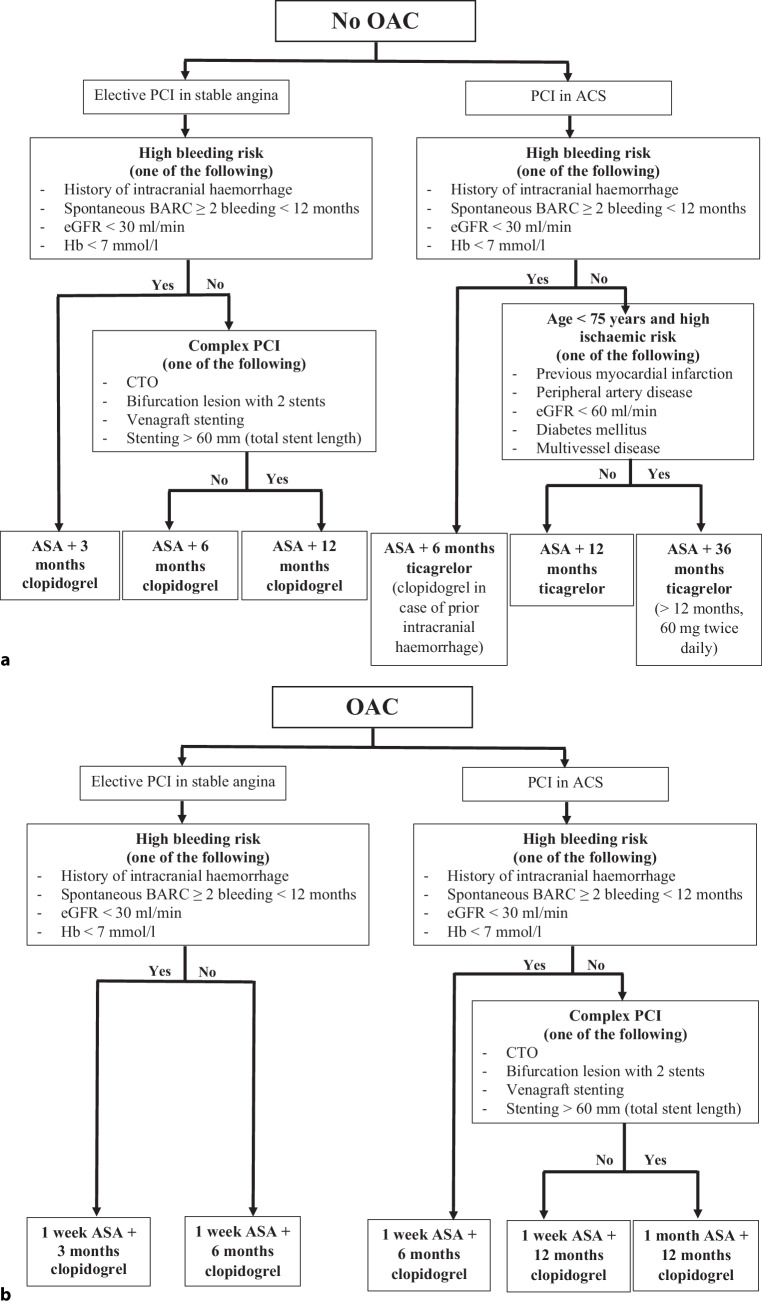
Protocols for personalised dual antiplatelet therapy in patients undergoing (*PCI*), for those **a** without concomitant oral anticoagulant (*OAC*) treatment and **b** with concomitant OAC treatment. *ACS* acute coronary syndrome, *BARC* Bleeding Academic Research Consortium, *eGFR* estimated glomerular filtration rate, *Hb* haemoglobin, *CTO* chronic total occlusion, *ASA* acetylsalicylic acid

All patients undergoing PCI are included in the registry. In line with the NHR, failed PCI attempts are considered as a performed PCI as long as a coronary wire has been inserted in the target vessel. Patients undergoing multiple PCIs are considered as a ‘new’ patient when the consecutive PCI is performed > 1 year after the index PCI. This is based on the follow-up duration of 1 year in the majority of patients and is in line with the NHR, in which a new 1‑year follow-up period is started for patients who present > 1 year after the initial PCI. Only in patients who are regarded to be at long-term high ischaemic risk based on their presentation and patient characteristics (Fig. 1), 2‑year follow-up data are collected. For all individual analyses, it will be determined and subsequently described whether a correction is required for patients who are included more than once in the registry.

All interim PCIs are regarded as follow-up to the index PCI. No in- or exclusion criteria are applied to achieve 100% registration in order to prevent bias. Therefore, the registry data can be regarded as real-world data.

### Periprocedural treatment and personalised antiplatelet therapy

The interventional procedures and concomitant pharmaceutical treatment are performed in accordance with the ESC Guidelines for CCS and ACS [9, 10]. To stimulate personalised secondary prevention post-PCI in accordance with the guidelines, the ZON-HR promotes the use of risk stratification after PCI to determine bleeding and ischaemic risks and guide antiplatelet strategy. To increase the feasibility of risk stratification, the ZON-HR Consortium has developed a simplified protocol for the antiplatelet strategy based on patient and procedural risk factors and the use of oral anticoagulants (Fig. 1). In this protocol, the criteria for bleeding and ischaemic risks are derived from the ESC Guidelines and are based on validated risk scores [2, 4, 8] and consensus documents, such as the criteria from the Academic Research Consortium for High Bleeding Risk [18]. The ZON-HR selected criteria for bleeding and ischaemic risks and excluded rare diseases or criteria commonly unknown at the time of the procedure. This protocol has been implemented at all participating centres where interventional cardiologists document the proposed antiplatelet strategy directly after PCI. The simplified protocol for bleeding risk has yet been externally validated and has shown a good prognostic value [19].

These criteria can be built into the electronic health record (EHR) and then generate an automated advice on antiplatelet duration according to the ZON-HR protocol. The interventional cardiologist can choose to follow or deviate from this advice according to the clinical setting.

To further enhance personalised treatment, the ZON-HR promotes active screening for *de novo* or undertreated DM by measuring HbA_1C_ values in patients admitted with ACS since 2022. In addition, monitoring of cholesterol management during out-patient visits is stimulated, specifically in ACS patients.

### Data collection

In addition to the NHR data, the ZON-HR collects additional risk factors at baseline for ischaemic and bleeding risks and data regarding procedural characteristics and periprocedural complications. Furthermore, follow-up data have been expanded by the addition of bleeding and ischaemic events and medication use. A full list of parameters included in this registry is presented in Table S1 (baseline characteristics) and Table S2 (follow-up characteristics) in the Electronic Supplementary Material. The definitions of these parameters are explained in the tables unless they have been previously described by the NHR [20, 21].

#### Baseline

At baseline, prespecified demographics, laboratory values and procedural characteristics (see Table S1 in Electronic Supplementary Material) that are available in the EHR are derived from the EHR and imported into Castor EDC. Additionally, medication at discharge standardly prescribed after a PCI or an ACS is collected in Castor EDC (see Table S1 in Electronic Supplementary Material). Discharge medication from referral centres is collected by digital questionnaires that are sent 1 week after discharge. Data regarding medical history, risk factors, complications during PCI and advice on the duration of dual antiplatelet therapy (DAPT) are registered by the interventional cardiologist that performed the PCI. These parameters are collected either directly in Castor EDC or in the EHR, from which they are exported and then imported into Castor EDC.

#### Follow-up and outcomes

Outcomes including complications after 1 month, 1 year and 2 years post-PCI (see Table S2 in Electronic Supplementary Material) are collected using digital questionnaires. Patient-reported outcomes are verified by EHR research and thereafter registered in the ZON-HR database.

Frequent monitoring of survey progression is done by researchers once per 1–2 weeks. If a patient fails to fill in the questionnaire, a reminder is sent. In case of no digital response, the patient is contacted by telephone in order to complete the follow-up. If this is not possible, the follow-up from non-responders is manually searched in the EHRs. This is required in approximately 60% of the patients. If these patients have their follow-up visits outside of the participating centres, they are regarded as lost to follow-up. Follow-up data collected by the NHR are matched with the data collected as described above in order to ensure completeness and consistency between the NHR and ZON-HR, which is of special importance for the follow-up of patients who do not receive follow-up treatment at one of the participating ZON-HR centres.

Laboratory results including LDL‑C and HbA1c values are collected from the EHR at 2 time points after PCI: at around 30 days and 365 days post-PCI. These questionnaires offer an opt-out option for the use of data for quality or research purposes. Approximately 3.5% of the patients who respond to the questionnaires choose the opt-out for the use of their data. In case of mortality, it is registered whether the cause of death was cardiovascular or non-cardiovascular. If the cause of death is uncertain, it is regarded as a cardiovascular death. For myocardial infarction, the universal definition is used [22], and bleeding events are defined according to the Bleeding Academic Research Consortium criteria [18]. Changes in antithrombotic medication are collected after 1 month, 1 year and 2 years by digital questionnaires. At 1‑year post-PCI, the use of cholesterol and DM medications is also collected. For non-responders, medication use is derived from the EHRs if available.

### Data protection and quality control

In Castor EDC, multiple tools are incorporated to guide data entry, such as settings for range and inserted explanatory texts or images. There is at least one study coordinator per site who has access to all records of this specific site in the EDC (study admin role). Furthermore, at least one study coordinator from each site is able to export the data of all sites. To reach a high degree of completeness, each centre can monitor their own data completeness in the EDC. Data are subject to the General Data Protection Regulation. In order to pseudonymise the data, no traceable personal data are stored in Castor EDC. Each site created and stored an identification log and stored it securely and separately at each site to retrace the patients back to the Castor identification number if required.

Data will be accessible to other researchers involved, such as interventional cardiologists from the participating centres, who can only add and view data of patients from their own site. A clinical event committee will periodically review 20% of the adverse events (randomly selected) to check whether all participating centres adhere to the same and the correct definitions of events. Furthermore, outcome differences between centres in the ZON-HR will be analysed to indicate possible gaps in the collection of certain outcomes in a centre. If one of the centres appears to monitor an outcome less well, extra follow-up checks within the EHR will be performed.

### Organisation

**Fig. 2:**
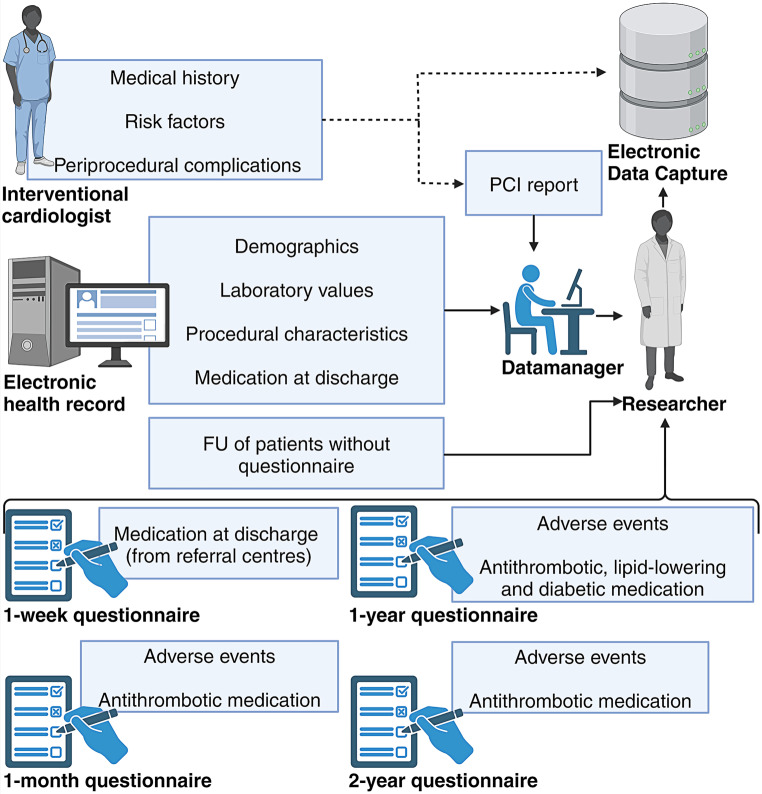
Workflow of South-East Netherlands Heart Registry*. PCI* percutaneous coronary intervention, *FU* follow-up (figure was created with BioRender.com)

The ZON-HR is led by a steering committee comprising representatives of the participating sites and a statistician. Weekly meetings are held by the executive committee, consisting of the principal investigators of the Radboud university medical centre and Maastricht University Medical Centre+ (R.J.M. van Geuns and A.W.J. van ’t Hof), interventional cardiologists and researchers from the participating sites. Student researchers are appointed to contribute to the collection of follow-up data. All proposals for research questions or research requiring pooled data are submitted to the steering committee and can be executed after approval. The workflow of the ZON-HR is graphically depicted in Fig. 2.

## Discussion

The ZON-HR is a contemporary, regional, multicentre, prospective PCI registry in the southeastern region of the Netherlands that collects data on medical history, risk factors at baseline, procedural characteristics, medication use and adverse events during follow-up. In addition, information about lipid and glucose metabolism is collected.

### Strengths

The ZON-HR provides extensive data that can be used for quality control and research purposes. This comprehensive registry represents the entire Dutch PCI population as it consists of both academic and non-academic hospitals and includes patients from the participating and referral hospitals. With the ZON-HR, we can visualise current gaps in the secondary prevention post-PCI and create awareness amongst treating physicians about treatment aspects that require attention. Analyses will be performed with data collected in a real-world setting, leading to more generalisable results. In addition, by promoting the use of risk stratification and by creating a simplified protocol for a personalised antiplatelet strategy based on the current ESC Guidelines, the ZON-HR aims to improve guideline-recommended medical treatment and prevention post-PCI in order to reduce adverse events.

The first research objective utilising this registry is to internally validate the simplified protocol for a personalised antiplatelet strategy in order to facilitate implementation elsewhere. We aim to demonstrate the predictive value of our stratification protocol for patients with high ischaemic and bleeding risk and determine the effect of adjusted DAPT duration according the ZON-HR protocol on outcomes. Furthermore, we aim to demonstrate adherence to the international guidelines on lipid-lowering therapy and the effect of cholesterol management on outcomes. Moreover, we aspire to improve secondary prevention in patients with established or *de novo* DM and demonstrate the effect of improved treatment on outcomes.

In addition to observational research, this registry offers the possibility for registry-based randomised clinical trials. The first study using the ZON-HR is the ReDUAL PCI Real Life Registry–based randomised controlled trial [23].

### Weaknesses

There are some weaknesses that are inherent to the registry study design. First, these patients are less frequently monitored compared with those in randomised clinical trials, resulting in a higher number of patients lost to follow-up. To mitigate the percentage that is lost to follow-up, non-responders to the questionnaires are approached by telephone.

Second, the collection of follow-up data using patient-reported outcomes is a weakness because this may lead to underreporting of events. On the other hand, in patients in whom follow-up data are collected by EHR research, adverse events that are reported elsewhere (e.g. by the general physician) are missed. Of note, patient-reported outcomes are always verified in the EHRs to ensure the reliability of outcomes.

The absence of a strict range for the dates by which follow-up must have been performed could also lead to underreporting of events as patients may not recall certain events after a period of time. However, by frequently monitoring survey progression, follow-up delay will be limited.

Lastly, medication use during follow-up is collected via questionnaires or the EHR. This may not accurately reflect patient adherence.

### Future perspectives

The ZON-HR aims to further improve personalised secondary prevention by providing further guidance on the treatment of risk factors. In addition to the personalised DAPT protocol, we aim to standardise screening and treatment for DM in the ACS population. Furthermore, we plan to create a personalised protocol on cholesterol management and implement this at the participating centres of the ZON-HR.

## Conclusion

The ZON-HR is a comprehensive PCI registry that provides baseline and follow-up data of a large PCI cohort in the southeastern region of the Netherlands. These data are used for quality control and research purposes. By this means, the ZON-HR aims to improve secondary prevention after PCI and augment personalised treatment that focusses on key elements of secondary prevention.

## Supplementary Information

*Table S1* Baseline characteristics collected in the ZON-HR

*Table S2* Follow-up outcomes collected in the ZON-HR
