# Supplementary material for: Design and rationale of the South-East Netherlands Heart Registry (ZON-HR)
Source: Neth Heart J. 2025 Feb 6;33(3):76–84. doi: 10.1007/s12471-025-01934-7 (PMC11845330; doi:10.1007/s12471-025-01934-7)
Supplement: Supplementary file 1 — Table S1 Baseline characteristics collected in the ZON-HR [file 12471_2025_1934_MOESM1_ESM.docx]

**Table S1** Baseline characteristics collected in the ZON-HR

| *Baseline parameters of the ZON-HR* | *NHR parameter* |
| --- | --- |
| *Patient characteristics* | |
| Date of intervention | yes |
| Age | yes |
| Sex | yes |
| Length (cm) | no |
| Weight (kg) | no |
| Lab values: Haemoglobin mmol/L; HbA1C mmol/mol; LDL-c mmol/L; creatinine µmol/l  *Most recent value within 3 months prior to PCI or 24 hours after PCI* | only creatinine |
| History of hypertension | yes |
| History of peripheral vascular disease | yes |
| History of spontaneous bleeding requiring medical attention (BARC ≥ 2)  If yes: Within the past 12 months prior to the PCI? | no |
| History of a cerebral vascular accident  If yes: Ischemic or haemorrhagic? | no |
| History of Diabetes Mellitus  If yes: no treatment; unknown treatment; insulin; diet/oral medication | yes |
| Left ventricular ejection fraction | yes |
| Dialysis dependent | yes |
| Smoking behaviour (regardless of quantity)  If previous smoking: Smoking in the past year? | no |
| Use of oral anticoagulants  If yes: Liver failure? Instable INR values? Alcohol use > 7 units/week? | no |
| Previous PCI | yes |
| Previous CABG | yes |
| PCI indication (CCS/NSTEMI/STEMI) | yes |
| Cardiogenic shock | yes |
| Out of hospital cardiac arrest | yes |
| *Procedural characteristics* | |
| Access site and second access site if required | yes |
| Treated vessel | yes |
| Type of intervention | yes |
| Multivessel disease | yes |
| Chronic total occlusion PCI (if yes, additional parameters are collected) | yes |
| 2-stent PCI of bifurcation lesion | no |
| Venagraft stenting | no |
| > 60mm stent (in total) | no |
| Stent diameter < 3 mm | no |
| Coronary perforation/dissection and use of covered stent | no |
| Hematoma/bleeding at access site | no |
| Pericardial effusion for which pericardial drainage is performed | no |
| Advice on DAPT duration (in months) by the interventional cardiologist | no |
| *Medication at discharge* |  |
| Aspirin, P2Y12 inhibitor; OAC; betablocker; ACE-inhibitor/angiotensin receptor blocker; statin; ezetimibe; PCSK9- inhibitors; Insulin; metformin; SGLT-2 inhibitors; proton pump inhibitors; NSAIDS | no |
| *ZON-HR* South East (Zuid Oost) Netherlands Heart Registration *NHR* Netherlands Heart Registration *INR* international normalized ratio *PCI* Percutaneous Coronary Intervention *CABG* Coronary Artery Bypass Grafting *CCS* Chronic Coronary Syndrome *(N)STEMI:* (non) ST Elevation Myocardial Infarction | |
