# Supplementary material for: Design and rationale of the South-East Netherlands Heart Registry (ZON-HR)
Source: Neth Heart J. 2025 Feb 6;33(3):76–84. doi: 10.1007/s12471-025-01934-7 (PMC11845330; doi:10.1007/s12471-025-01934-7)
Supplement: Supplementary file 2 — Table S2 Follow-up outcomes collected in the ZON-HR [file 12471_2025_1934_MOESM2_ESM.docx]

**Table S2** Follow-up outcomes collected in the ZON-HR

| *Follow-up parameter of the ZON-HR* | *NHR parameter* |
| --- | --- |
| *Adverse events (including dates)* | |
| Bleeding  If yes: BARC score; access or non-access site; spontaneous or not | no |
| Cerebral vascular accident  If yes: Ischemic or haemorrhagic? | no |
| Stent thrombosis  If yes: *Definite* (angiographic or pathologic confirmation)/  *Probable* (unexplained death in 30 days or TVR without angiographic confirmation) | yes  (only definite within the first year) |
| Myocardial infarction  If yes: Within or after 48 hours from current procedure/  Within 48 hours after re-intervention (PCI or CABG) | yes/no  (only within 30 days) |
| CABG (all)  If yes: TVR/TLR/different vessel. Staged or non-staged | yes/no  (only within 24 hours) |
| PCI  If yes: TVR/TLR/different vessel. Staged or non-staged | yes  (only within the first year) |
| Death  If yes: Cardiovascular or non-cardiovascular mortality | yes  (not specified) |
| *LDL-C values (including date of measurement)* | |
| 30 days after PCI (range 30-90 days, value closest to 30 days)  365 days after PCI (range 335-445 days, value closest to 365 days) | no |
| *Medication use* | |
| 30 days after PCI: Aspirin, P2Y12 inhibitors; oral anticoagulants  1 year after PCI: Aspirin, P2Y12 inhibitors; oral anticoagulants; statins; ezetimibe; PCSK-9 inhibitors; In patients with DM: Insulin; metformin; SGLT-2 inhibitors  2 years after PCI: Aspirin, P2Y12 inhibitors; oral anticoagulants | no |
| *ZON-HR* South East (Zuid Oost) Netherlands Heart Registration *NHR* Netherlands Heart Registration *BARC* Bleeding Academic Research Consortium *TVR* Target Vessel Revascularisation *PCI* Percutaneous Coronary Intervention *CABG* Coronary Artery Bypass Grafting *LDL-C* Low Density Lipoprotein Cholesterol | |
